# Optimisation and characterisation of the orange pigment produced by a cold adapted strain of *Penicillium* sp. (GBPI_P155) isolated from mountain ecosystem

**DOI:** 10.1080/21501203.2017.1423127

**Published:** 2018-01-09

**Authors:** Neha Pandey, Rahul Jain, Anita Pandey, Sushma Tamta

**Affiliations:** a Biotechnological Applications, G.B. Pant National Institute of Himalayan Environment and Sustainable Development, Almora, Uttarakhand, India; b Department of Botany, DSB campus, Kumaun University, Nainital, Uttarakhand, India

**Keywords:** *Penicillium*, carotenoids, LC/MS, FT-IR, antimicrobial activity, Indian Himalayan region

## Abstract

With globalisation and growing health risks of synthetic colourants, search for pigments from natural sources has increased owing to their non-toxic nature. The present study highlights the bioprospection of natural pigment from a cold adapted fungal strain of *Penicillium* sp. (GBPI_P155), isolated from soil of Indian Himalayan region. The fungus produced insoluble and orange-coloured pigment in liquid medium with maximum production recorded in potato dextrose (PD) broth at 15°C and 3 pH, while maximum biomass was produced at 25°C and pH 3. While examining the effect of different mineral salts, and carbon and nitrogen sources on pigment production, maximum accumulation of pigment was recorded in PD broth supplemented with 2% maltose. Following production, extraction of pigment was performed using chloroform and characterised partially by UV/vis (*λ*_max_ at 495 nm and a shoulder peak at 530 nm) and Fourier Transform Infrared (FT-IR) spectroscopy. Thin layer chromatography of chloroform extract resulted in separation of pigment in three fractions with Rf values 0.911, 0.852 and 0.808, which were further analysed using Liquid Chromatography Mass Spectrometry (LC/MS). The overall approach resulted in identification of pigment as a mixture of different derivatives of carotenoids. The extracted pigment also possessed antimicrobial activity against Gram-positive and Gram-negative bacteria and actinobacteria.

## 1. Introduction

Cold adapted fungi are increasingly getting recognition due to their ecological and biotechnological significance. These fungi secret extraordinary compounds of commercial applications which include novel enzymes, antibiotics, feed products, pigments etc. (Ferreira et al. 2016; Wang et al. 2017). A wide range of pigments is produced by these fungi representing several chemical classes such as carotenoids, melanins, flavins, phenazines, quinones, and more specifically monascins, violacein or indigo. Generally, these pigments are the secondary metabolites and are grouped into four classes namely terpenes, polyketides, non-ribosomal peptides and amino acid-derived compounds that possess different biosynthetic pathways (Studt et al. 2012). Ascomycetous fungi have a great potential to produce pigments which can be used as an alternative to synthetic pigments. With increasing consumer interest towards the use of natural sources for dying and food colourant purpose, demand for natural pigments has considerably grown over synthetic counterparts (Ganesh et al. 2011). Most studied ascomycetous filamentous fungi known to produce pigments belong to genera *Talaromyces, Trichoderma, Aspergillus, Fusarium, Monascus, Neurospora* and *Penicillium* (Dufosse et al. 2014; Gmoser et al. 2017).

Pigments are generally produced in the cell cytoplasm as a response to adverse environmental conditions (Pagano and Dhar 2015), vis-à-vis have ecological functions. Different pigments help in improving the survival of fungi, for example, carotenoids protect against harmful ultraviolet radiation and light (lethal photo-oxidation). Similarly, melanins and secondary carotenoids, such as canthaxanthin and astaxanthin tend to accumulate in response to environmental stress (Bhosale 2004). Moreover, secondary metabolites such as statin, naphthoquinones and carotenoids produced from microorganisms possess anitimicrobial, antioxidant and anticancer activities (Kumaresan et al. 2008). Astaxanthin is reported to possess bioactivity against *Helicobacter pylori* (Kirti et al. 2014).

Indian Himalayan region (IHR), being one of the hot spots of biodiversity along with having extreme climatic conditions, offers a huge potential for isolation of unique microbiota possessing rich biotechnological applications. Diversity of psychrotolerant fungi in this region has been explored time to time. A unique diversity of *Penicillium* species having extremophilic characteristics, isolated from Indian Himalayan soils, has been reported by Dhakar et al. (2014b). *Penicillium*, being a dominant fungal genera in Indian Himalayan soils, has been reported to possess several applications e.g. phosphate solubilisation, biodegradation, enzyme production etc. (Pandey et al. 2008, 2016). Continuous and higher production of metabolites at suboptimal conditions has been an exclusive feature observed among these fungi (Dhakar et al. 2014a). Continuing harnessing this dominant genera, the present study emphasises on an orange pigment producing *Penicillium* sp. focusing on the extraction and characterisation of the pigment. Antimicrobial activity against bacteria, actinobacteria and fungi has also been examined.

## 2. Materials and methods

### 2.1. Fungal isolate (GBPI_P155) and its characterisation

The fungal isolate (GBPI_P155), producing dark orange pigment, was initially isolated from high altitude soil of IHR (Chaurasia et al. 2005). Methodology used for morphological, microscopic and physiological characterisation of GBPI_P155 has been described in previous studies (Dhakar et al. 2014b; Pandey et al. 2016). Identification of the fungal isolate was done by amplifying ITS region (ITS1-5.8S-ITS2) using PCR and sequencing of the amplified product was performed in collaboration with National Centre for Cell Science, Pune, India. GBPI_P155 was maintained on Potato Dextrose (PD) agar slants and preserved at 4°C. Sub-culturing of the fungal isolate was done on PD agar, incubating at 25°C for 5–7 days before performing any experiment.

### 2.2. Pigment production and optimisation

**Figure 1.**
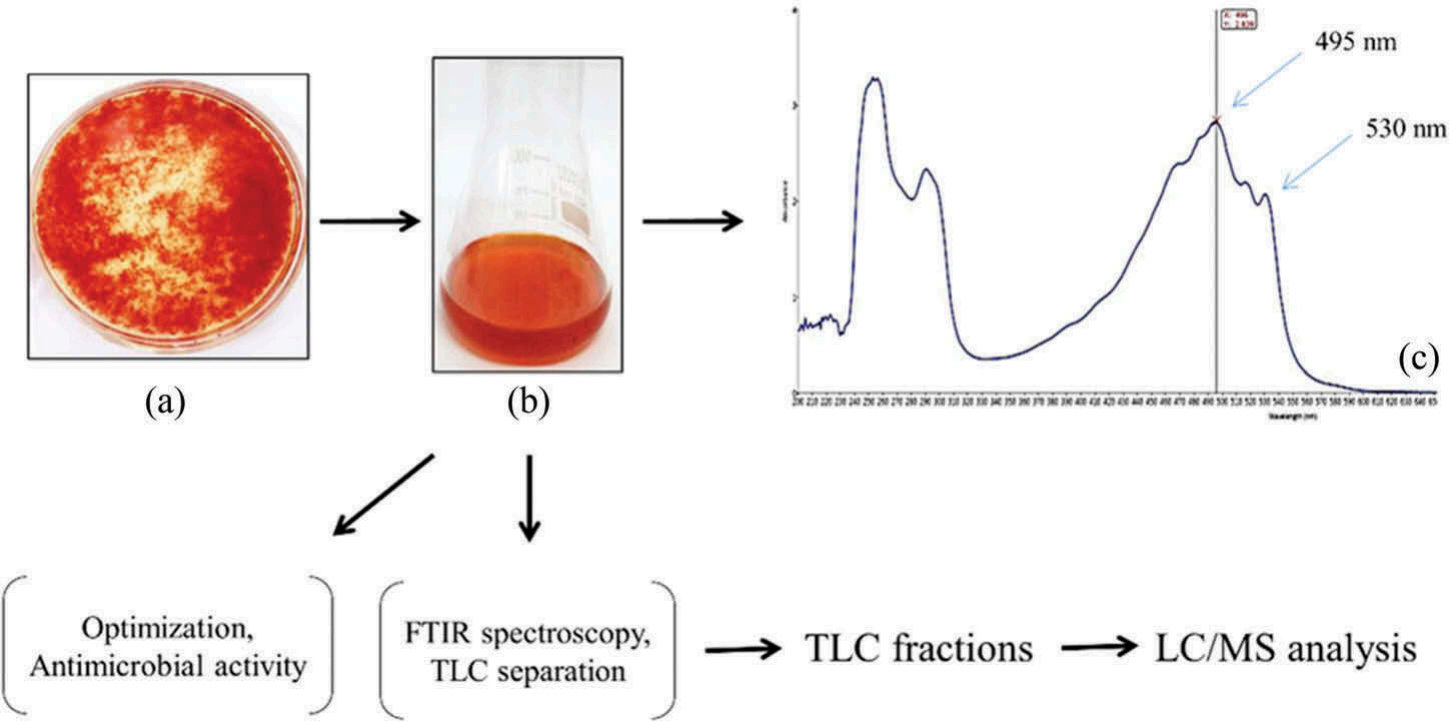
Scheme of GBPI_P155 pigment characterisation.

An overview of the scheme utilised for pigment production from GBPI_P155 is shown in Figure 1.

#### 2.2.1. Pigment production by GBPI_P155 in different medium

Five different broths including PD broth, malt extract (ME) broth, Sabouraud Dextrose broth (SD), nutrient broth and Czapek Dox (CD) broth were considered for production of pigment by GBPI_P155. A 5 mm disc (~6 × 10^8^ spores/mL) from a freshly grown fungal colony (25°C for 5 days on PD agar) was inoculated in 250 mL Erlenmeyer flask, each containing 50 mL sterilised broth. The production of orange pigment in different medium was examined on the basis of visual inspection.

#### 2.2.2. Effect of temperature and pH on pigment production

All the optimisation experiments were carried out in PD broth unless otherwise mentioned. For determining optimum temperature requirement for pigment production, 50 mL broth containing fungal disc was incubated at different temperatures (5, 15, 25 and 35°C). Similarly, optimum pH was determined after the pH of the medium was adjusted to pH 2, 3, 5, 7, 9, 11 and 13, and the broth was inoculated with 5 mm fungal disc and incubated at optimum temperature under static condition. Pigment production was measured at every 5th day and up to 30 days. Pigment production in broth was quantified based on the characteristic lambda max of orange pigment using UV/vis spectrophotometer after extraction with equal volume of chloroform.

#### 2.2.3. Effect of carbon and nitrogen sources

Influence of different carbon supplements (2%) including glucose, fructose, lactose, maltose, and sucrose and nitrogen supplements (1%) including peptone, yeast extract, sodium nitrate and sodium nitrite on pigment production was determined. A disc of freshly grown fungal culture of GBPI_P155 was inoculated to the PD broth supplemented with different carbon and nitrogen sources and incubated at 25°C for 30 days in static condition. Pigment production at every 5th day was recorded as described earlier.

#### 2.2.4. Effect of mineral salts

An amount of 50 mL broth was supplemented with 0.5% of different salts including magnesium sulphate (MgSO_4_), zinc sulphate (ZnSO_4_), copper sulphate (CuSO_4_), dihydrogen potassium phosphate (KH_2_PO_4_) and ferric chloride (FeCl_2_). Fungal disc was inoculated in the mineral salt supplemented medium and incubated at optimum temperature for 30 days. Pigment production at every 5th day was recorded as previously described.

### 2.3. Biomass estimation

Fungal biomass was determined by collecting mycelium after filtration of fungal broth using Whatman No. 1 filter paper. Mycelium was washed and dried at 55°C until complete dryness or till constant weight was not obtained. The weight of dried mycelium was measured (Pandey et al. 2016).

### 2.4. Characterisation and identification of the pigment produced by GBPI_P155

For characterisation of orange pigment, 15-day-old culture broth (25°C) was taken in a separating funnel after removal of fungal mycelium. To this, equal volume of chloroform was added and pigment was extracted in organic phase after proper mixing. Chloroform separated part was subjected to scanning in a UV/vis spectrophotometer and characteristic lambda max of the pigment was recorded. Chloroform-extracted pigment was air dried and dissolved in minimum volume of chloroform for further studies. Functional group analysis in extracted pigment was performed using Cary 630 FT-IR spectrometer (Agilent Technologies) in spectral range from 650 to 4000 cm^−1^ and at a spectral resolution of 8 cm^−1^. The spectrum obtained was subjected to analysis for presence of possible functional group based on the wave numbers.

Thin layer chromatography (TLC) (silica gel 60 F_254_, Merck, Germany) using hexane:acetone:toluene:ethanol (10:7:7:6) solvent system as mobile phase (Delgado-Vargas et al. 2000) was performed. The Rf value of separated pigment was recorded and resulting spots were scraped from the TLC plate and eluted in methanol. The eluted spots were used for identification studies. The methanol eluted pigment fractions were further subjected to LC/MS analysis (MS Synapt GZ HDMS LC-MS UPLC H-Class) using 0.1% formic acid:acetonitrile:methanol as mobile phase with column temperature of 35°C and having C18 column (1.7 µm) type. The chromatogram and mass spectrum were analysed and compared with the standard mass spectrum of Metlin library for identification of the compounds.

### 2.5. Antimicrobial activity of chloroform extracted pigment

Antimicrobial activity of dried chloroform-extracted pigment against bacteria, actinobacteria and fungi was performed using disc diffusion assay. The dried extract was prepared in four different solvents (ethyl acetate, chloroform, butanol and methanol). A 24 h grown bacterial culture and 5-day-old actinobacterial and fungal cultures were evenly spread on agar plates (TY for bacteria and actinobacteria and PD for fungi). 20 μl of dissolved extract was loaded on 5 mm sterilised disc of Whatman filter paper 1 kept over agar surface. Plates were then incubated at 25°C for 24 h for bacteria and 5 days for actinobacteria and fungi. Control for each organic solvent and for each test organism was also taken simultaneously to nullify the inhibition due to solvent. Zone of inhibition was calculated after incubation as described in Jain and Pandey (2016).

## 3. Results

### 3.1. Characterisation and identification of GBPI_P155

**Figure 2.**
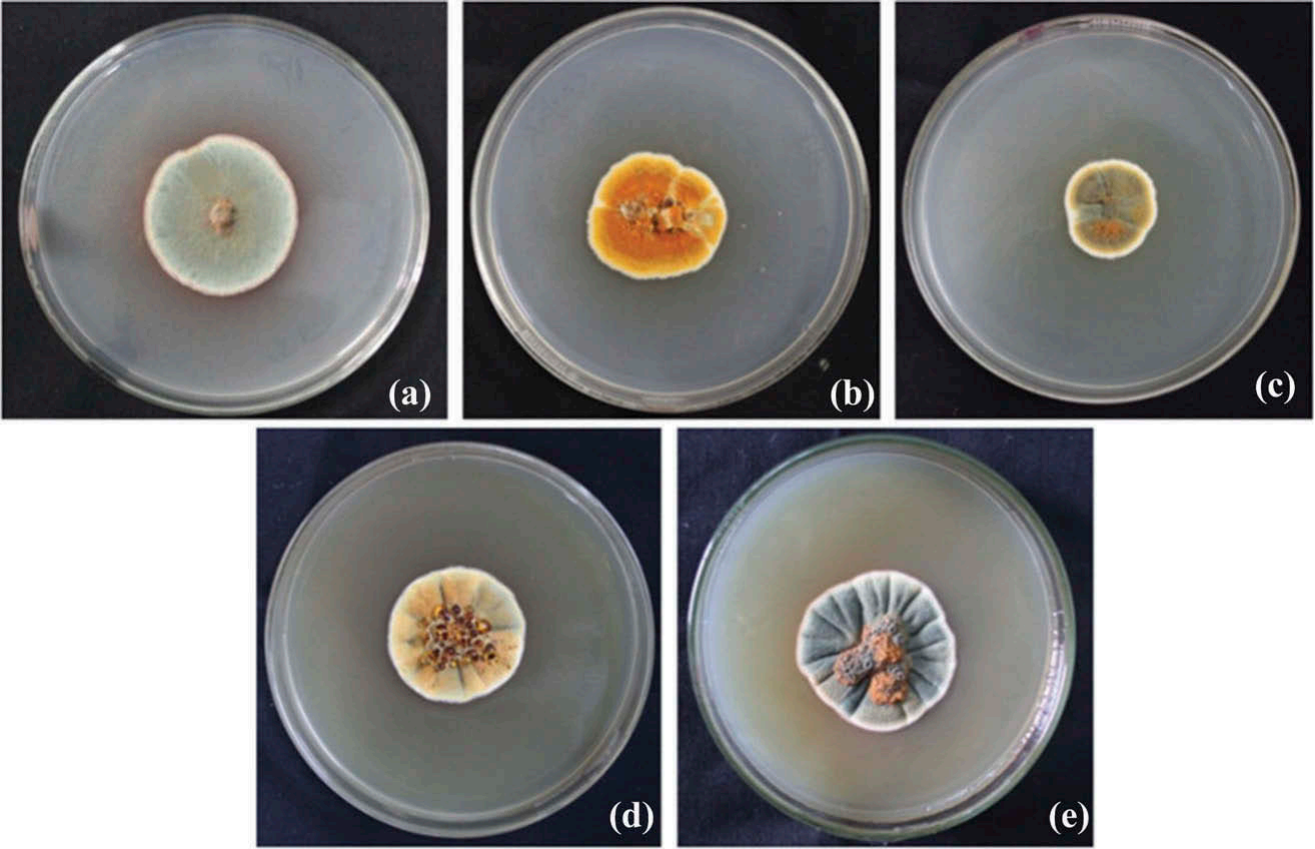
Morphology of GBPI_P155 fungus on different agar medium (a) Potato Dextrose, (b) Czapeck Dox, (c) Nutrient agar, (d) Malt extract and (e) Sabouraud Dextrose.

The fungal isolate GBPI_P155 produced colonies having white margins with green sporulation releasing orange exudates on PD agar plate after 5 days of incubation at 25°C. Morphology on different agar medium was also recorded which is shown in Figure 2. Microscopic observation of GBPI_P155 revealed presence of branched conidiophores. The fungal isolate showed growth at temperature ranging from 5 to 35°C with the optimum growth at 25°C. The growth was observed at pH ranging from 2 to 14 (optimum 5–7), and salt concentration up to 12%. Identification of the fungus, on the basis of the ITS region analysis, showed maximum similarity with *Penicillium* sp. Nucleotide sequence (accession number: KC634225) and culture (accession number: MCC1061) have been deposited in the GenBank NCBI and National Centre for Microbial Resource, NCCS, Pune, India, respectively.

### 3.2. Pigment production and optimisation

Amongst the five medium tested for pigment production, maximum pigmentation in the form of water insoluble precipitate was observed in PD broth at optimum growth temperature. Therefore, PD broth was considered for performing further optimisation experiments.

#### 3.2.1. Effect of temperature and pH

**Figure 3.**
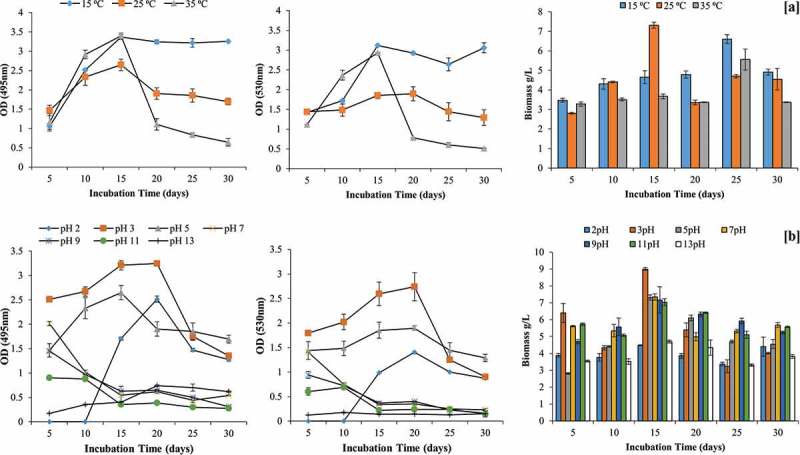
Effect of temperature and pH on pigment production by GBPI_P155 and biomass estimation.

Pigment production was observed at temperature ranging from 15 to 35°C with maximum production recorded at 15°C (OD_495_ = 3.37; OD_530_ = 3.12) and 35°C (OD_495_ = 3.39; OD_530_ = 2.93) in 15 days of incubation. No further increment in pigment production was recorded after 15 days at 15°C. Pigmentation was not observed in PD broth at lower temperature (below 10°C). In contrast to pigment, biomass production was found to be maximum at 25°C (7.31 ± 0.15 g/L) in 15 days of incubation as shown in Figure 3(a).

Likewise temperature, pH also influenced pigment production. Maximum pigment was produced in acidic medium i.e. pH 5 (OD_495_ = 3.24; OD_530_ = 2.74) on 20th day of incubation, followed by pigment production at pH 5 (OD_495_ = 1.9; OD_530_ = 1.89). No pigment production was observed in pH range from 7 to 13. Maximum biomass was also recorded at 3 pH (9.00 ± 0.08 g/L) on 15th day of incubation (Figure 3(b)).

#### 3.2.2. Influence of carbon and nitrogen sources and mineral salts

**Figure 4.**
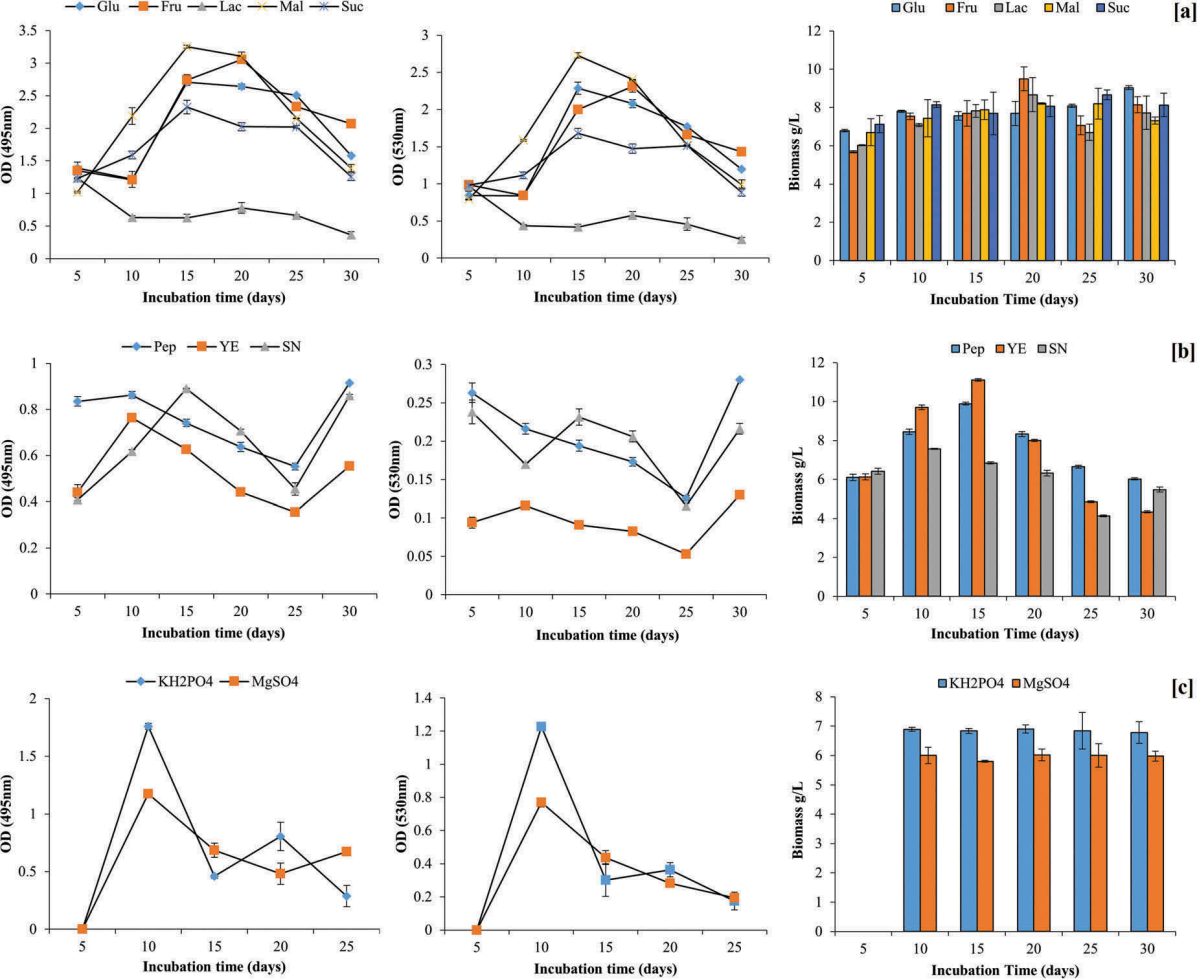
Effect of carbon and nitrogen source and mineral salts on production of pigment by GBPI_P155 and simultaneous biomass estimation.

Additional maltose as C source in the PD broth enhanced pigment production (OD_495_ = 3.25; OD_530_ = 2.73) in comparison to control medium having only PD broth. This was followed by fructose and glucose. In contrast, lactose, as additional C source, inhibited the orange pigment production by GBPI_P155. Contrary to pigment production, maximum biomass was recorded in presence of fructose (9.50 ± 0.62 g/L) on 20th day of incubation followed by lactose (8.67 ± 0.89 g/L) on same day (Figure 4(a)).

Overall, nitrogen supplementation in the PD broth inhibited the pigment production. Among the tested N source, peptone supported maximum pigment production on 10th day (OD_495_ = 0.86) and 30th day of incubation (OD_495_ = 0.91). In contrast to this, maximum biomass (11.11 ± 0.07 g/L) was observed in yeast extract-supplemented medium on 15 days of incubation. No fungal growth and pigment production were observed after supplementing the medium with sodium nitrite (Figure 4(b)).

Among different mineral salts tested, only KH_2_PO_4_ and MgSO_4_ were able to facilitate pigment production by GBPI_P155. Maximum pigment production in presence of KH_2_PO_4_ (OD_495_ = 1.76; OD_530_ = 1.23) and MgSO_4_ (OD_495_ = 1.17; OD_530_ = 0.77) was observed on day five of incubation. However, the biomass produced in the presence of MgSO_4_ was found to be maximum in 25 days of incubation followed by KH_2_PO_4_, whereas other mineral salts such as Zn^2+^, Cu^2+^ and Fe^2+^ showed restricted biomass (Figure 4(c)).

### 3.3. Extraction, characterisation and identification of pigment

**Figure 5.**
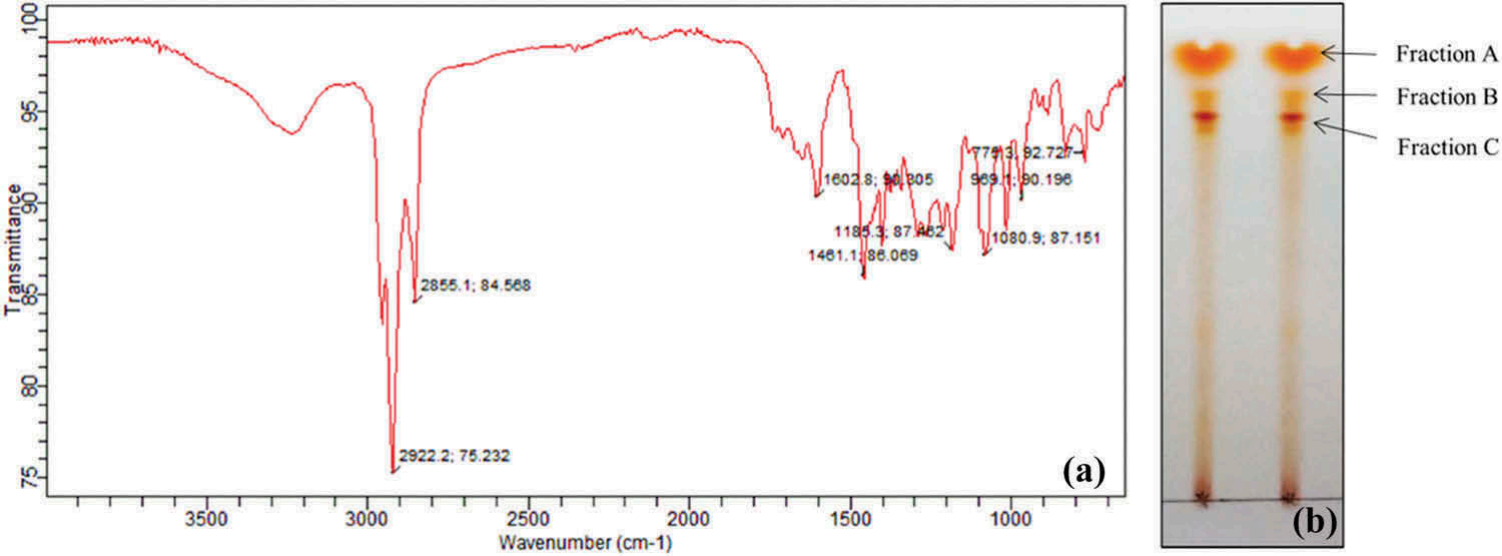
(a) FT-IR spectrum and (b) TLC separation of chloroform extracted pigment produced by GBPI_P155.

Although various solvents were examined for the extraction of pigment, chloroform was found to be the most suitable for extraction purpose. Scanning under UV–vis spectroscopy resulted in two major absorption peaks at 495 nm and a shoulder peak at 530 nm. FT-IR analysis of chloroform extracted pigment was performed to identify bonding molecules. The main absorbance peaks detected in IR spectrum included 2922.2, 2855.1, 1602.8, 1461.1, 1185.3 and 1080.9 cm^−1^ (Figure 5(a)). The peaks at 2922.2 and 2855.1 cm^−1^ were moderately sharp, indicating possible presence of C–H group. Peak with wave number of 1602.8 cm^−1^ showed presence of C=C group. On the other hand, spectral peak at 1461.1, 1185.3 and 1080.9 cm^−1^ suggested the presence of CH_3_ and C–O group.

**Figure 6.**
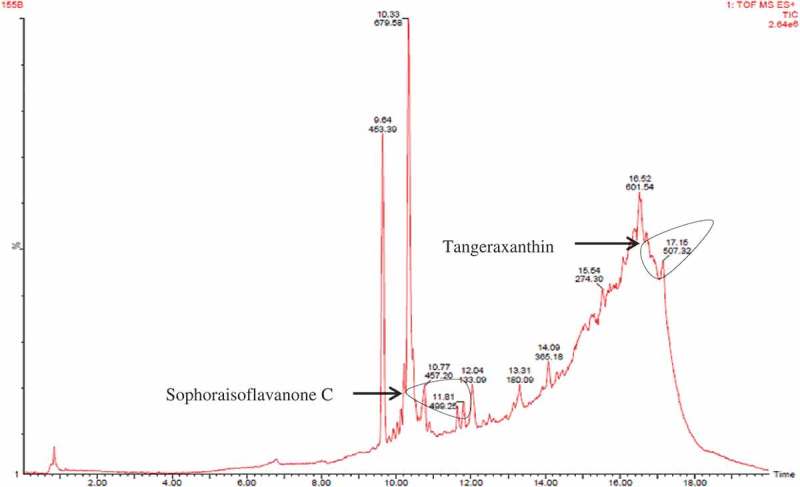
LC/MS analysis of fraction A.

Chloroform-extracted pigment was separated using TLC. Three bands were observed in the TLC having Rf values of 0.91, 0.85 and 0.81 (Figure 5(b)). These three bands were eluted and dissolved in methanol and stored in Eppendorf tube individually. Three factions (fraction A, B and C) were filtered through 0.45 µm membrane filter and analysed using LC/MS for identification of possible compounds. Fraction A contained derivative of carotenoid named tangeraxanthin having mass 507.32 ([M + Na]^+^) and a derivative of flavonoid namely sophoraisoflavoanone C having mass 499.25 ([M + Na]^+^) along with several other compounds as shown in Figure 6 and Table S1.

**Figure 7.**
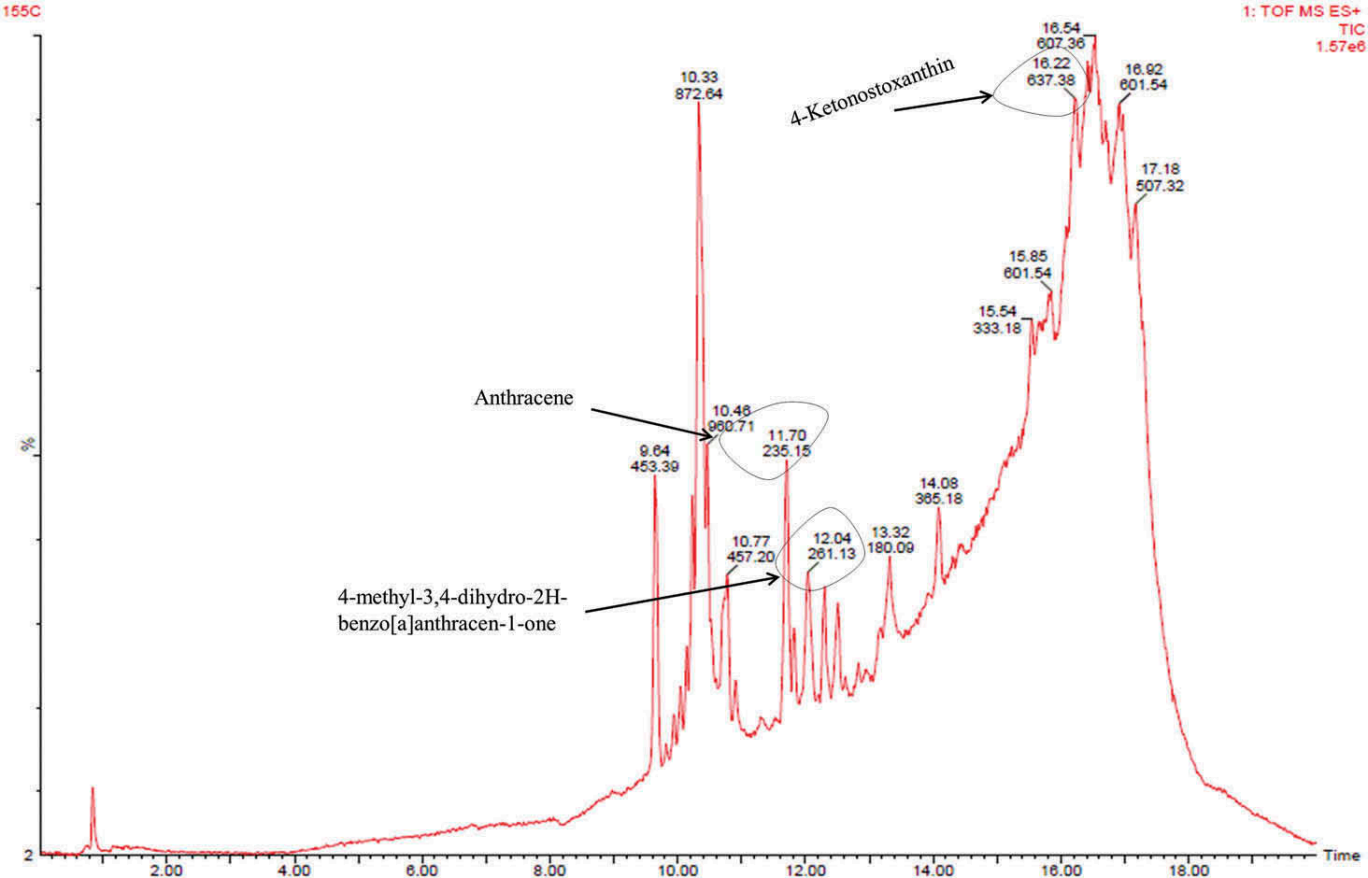
LC/MS analysis of fraction B.

Fraction B showed presence of derivatives of anthracene i.e. anthracene, 2,3,9,10-tetramethyl and 4-methyl-3,4-dihydro-2*H*-benzo[*a*]anthracen-1-one exhibiting mass 235.15 and 261.13 ([M + H]^+^), respectively. Carotenoid derivative (tangeraxanthin and 4-ketonostoxanthin with mass 507.32 and 637.38 ([M + Na]^+^), respectively), along with other compounds, was also identified in fraction B as shown in Figure 7 and Table S2.

**Figure 8.**
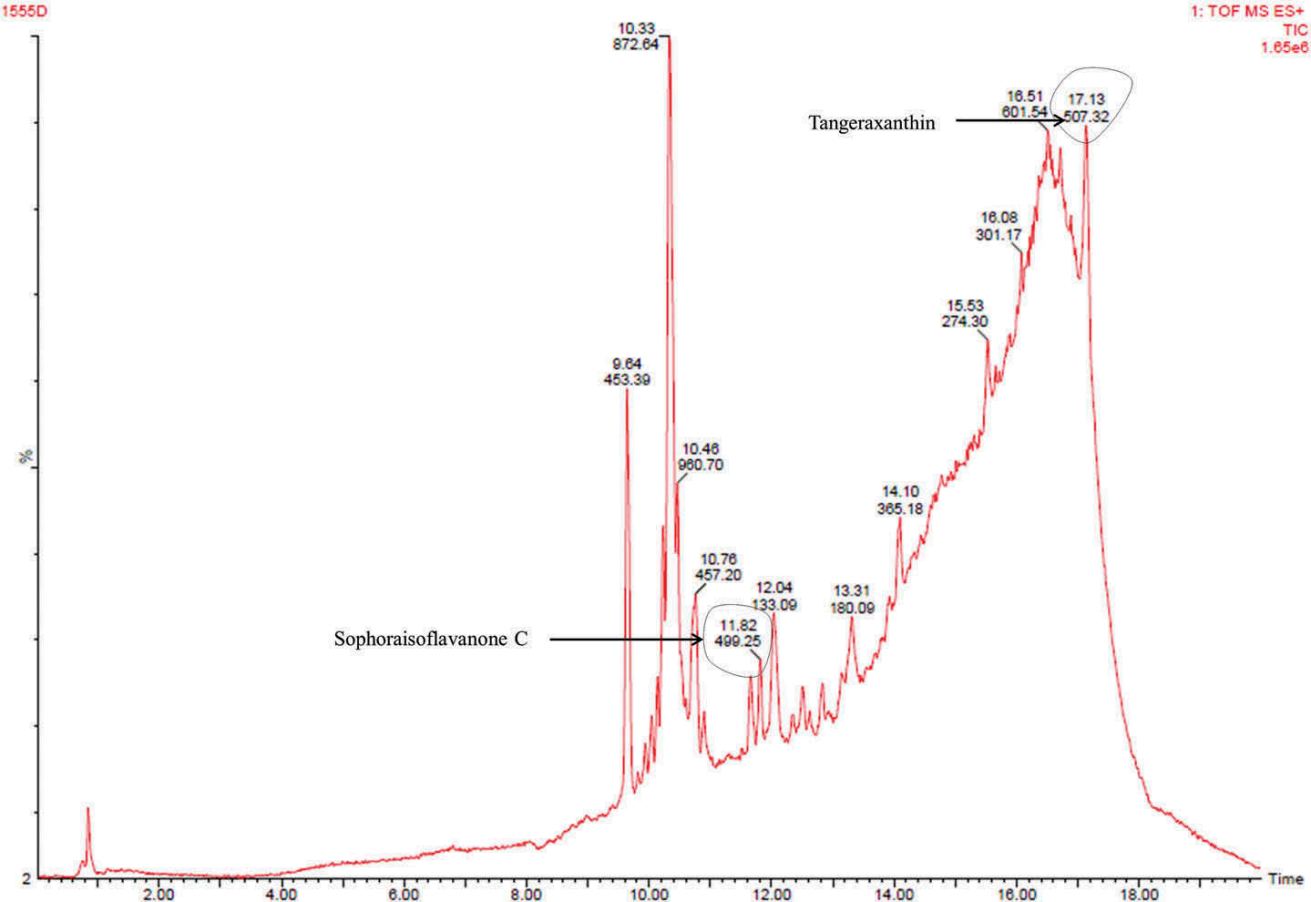
LC/MS analysis of fraction C.

Third fraction (fraction C) contained compounds (tangeraxanthin and sophoraisoflavanone C) similar to the fragment A with some other compounds as shown in Figure 8 and Table S3. Therefore, LC/MS analysis indicated presence of derivatives of carotenoid as major pigment, and anthracene and flavonoid as minor component in the pigment extracted from the psychrotolerant *Penicillium* sp. (GBPI_P155).

### 3.4. Antimicrobial activity

**Figure 9.**
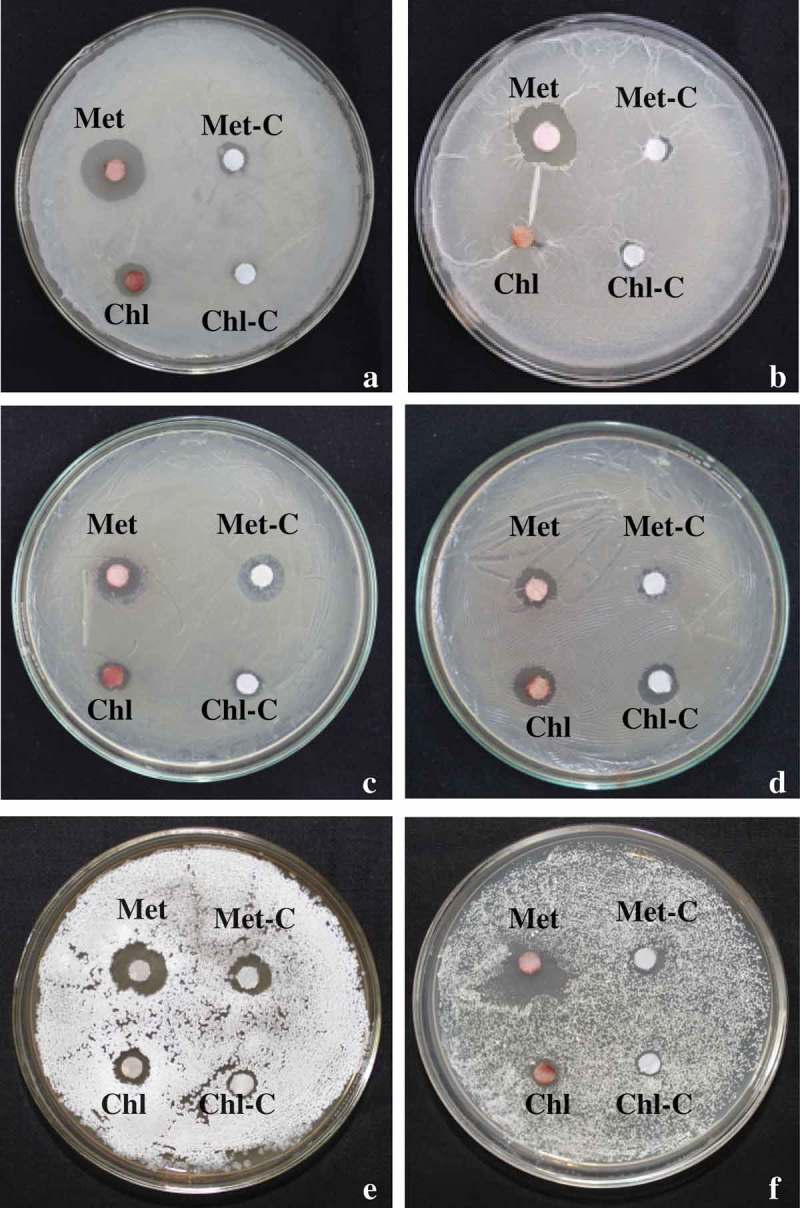
Antimicrobial activity of dried pigment in methanol (Met) and chloroform (Chl). (a) *Bacillus subtilis*, (b) *Bacillus megaterium*, (c) *Pseudomonas putida*, (d) *Pseudomonas marginalis*, (e) *Streptomyces* sp. and (f) *Nocardia tenerifensis*; C: control.

Antimicrobial assays were performed against Gram-positive (*Bacillus megaterium, Bacillus subtilis*) and Gram-negative bacteria (*Serratia marcescence, Escherichia coli, Pseudomonas putida* and *Pseudomonas marginalis*), actinobacteria (*Nocardia tenerifensis, Streptomyces* sp.) and fungi (*Alternaria alternata, Phytophthora* sp., *Fusarium solani* and *Fusarium oxysporum*). Maximum inhibition zone of 9.50 and 8.50 mm in methanol was recorded against Gram-positive *B. megaterium* and *B. subtiliis*, respectively. In case of Gram-negative bacteria, maximum inhibition zone of 1.6 mm for *S. marcescens* in chloroform, 0.80 mm for *E. coli* in butanol, 3.60 mm for *P. putida* and 2.50 mm for *P. marginalis* in methanol was recorded. Extracted pigment dissolved in methanol also showed inhibition zone against actinobacteria (2.50 mm for *N. tenerifensis* and 3.60 mm for *Streptomyces* sp.). In contrast to this, no antimicrobial activity was observed against fungal pathogens. The antimicrobial activity of pigment dissolved in different solvents against Gram-positive (*Bacillus* spp.), Gram-negative (*Pseudomonas* spp.) bacteria and actinobacteria is shown in Table 1 and Figure 9.

**Table 1.** Antimicrobial activity of pigment against bacteria and actinobacteria.

S. No.	Microorganism	Organic solvents			
		Methanol	Chloroform	Ethyl acetate	Butanol
		(Inhibition zone [mm] ± SE)			
1	*Serratia marcescence*	0.8 ± 0.16	1.6 ± 0.33	0.8 ± 0.16	NA
2	*Bacillus megaterium*	9.5 ± 0.28	NA	2.5 ± 0.28	1.5 ± 0.29
3	*Bacillus subtilis*	8.5 ± 0.29	1.5 ± 0.28	6.6 ± 0.30	2.6 ± 0.33
4	*Escherichia coli*	NA	NA	NA	0.8 ± 0.16
5	*Pseudomonas putida*	3.6 ± 0.33	0.8 ± 0.16	2.6 ± 0.33	2.5 ± 0.28
6	*Pseudomonas marginalis*	2.5 ± 0.28	0.8 ± 0.16	1.6 ± 0.33	0.8 ± 0.16
7	*Nocardia tenerifensis*	2.5 ± 0.28	NA	NA	NA
8	*Streptomyces* sp.	3.6 ± 0.33	3.5 ± 0.29	1.5 ± 0.29	2.5 ± 0.29

mm: Millimeter; SE: standard error; NA: no activity.

## 4. Discussion

In the present investigation, the fungal isolate GBPI_P155 identified as *Penicillium* sp. was studied for the production of orange pigment. Ascomycetous fungi including *Monascus, Penicillium, Fusarium, Aspergillus, Paecilomyces* etc. are generally known for production of various bioactive compounds including enzymes and pigments. Several enzymes are responsible for the synthesis of pigments, generally by the mevalonate and polyketide pathways (Mapari et al. 2010; Afshari et al. 2015). GBPI_P155 possessed (poly)extremophilic characters as fungus tolerated a wide range of temperature and pH and high salt concentration. Studies on wide pH tolerant microorganisms from Indian Himalayan soil have been reported and reviewed as well (Pandey et al. 2001; Rinu and Pandey 2010; Dhakar and Pandey 2016).

PD broth was found to be a well-suited medium for the production of orange pigment by GBPI_P155. Lebeau et al. (2017) also reported the highest production of a red pigment by the strain of *Penicillium purpurogenum rubisclerotium* grown in PD broth medium, incubated at 26°C, whereas biomass concentration was found to be low in this medium. Similar results were obtained in the present study where maximum pigmentation was recorded after 15 days of incubation. Longer incubation is generally required for production of such secondary metabolites from filamentous fungi (Afshari et al. 2015). In order to achieve maximum pigment yield, optimisation of temperature and pH is always crucial as they influence the physiological activity and metabolic growth of the organisms. Being a cold-tolerant fungus, temperature played a vital role in pigment production. GBPI_P155 produced higher amount of pigment at 15°C in comparison to optimum growth temperature of 25°C, though maximum biomass production was recorded at 25°C. Pigment production at low temperature indicated towards the phenomenon of ecological resilience amongst psychrotolerant *Penicillium* spp. to survive the stress environments. Also, increase in pigment production below optimal temperature can be suggested as an acclimating response compensating for down regulation of metabolic process at lower temperatures (Gmoser et al. 2017). Observations on the similar lines with respect to ascomycetous fungi have been reported from our laboratory. For example, exposure to low temperature (4°C) induced heavy sporulation in three psychrotolerant species of *Trichoderma* (*T. harzianum, T. konengii, T. viride*) initially isolated from glacial sites in IHR (Ghildiyal and Pandey 2008). Similarly, a psychrotolerant and phosphate solubilising fungus (*Paecilomyces hepiali* MTCC 9621), isolated from cold desert Himalaya, solubilised maximum phosphate at 14°C (Rinu and Pandey 2011; Rinu et al. 2012). Prolonged or over production of secondary metabolites while appeared as one of the survival strategies possessed by these cold-adapted fungi for combating low temperature stress, the phenomenon indeed extend unlimited opportunities for biotechnological applications.

Low optimum pH (pH 3) for pigment as well as biomass production by GBPI_P155 could be supported through several studies which suggest pigment production by fungi in acidic conditions during submerged culture (Cho et al. 2002). Low pH inhibits the conidia development and enhances pigment production, which indicates that media pH influences transport of certain media constituents (Lee et al. 2001). Also, pH can affect enzyme activity involved in biosynthesis of pigments (Pisareva et al. 2005). Optimum red pigment production by *P. purpurogenum* GH2 was observed at 24°C and pH 5 in CD medium with no relation between pigment and biomass production (Mendez et al. 2011). Afshari et al. (2015) reported that *Penicillium aculeatum* ATCC 1049 produces yellow pigment at 30°C and pH 6.5 while the biomass (11.12 g/L) production was found to be maximum at 30°C and pH 8. Chen and Johns (1993) observed that *Monascus purpureus* produces maximum yellow pigment at pH 4. Similarly, optimum yellow pigment production was obtained at pH between 3 and 3.5 in *Monascus* (Babitha et al. 2007; Hernández-Rivera et al. 2008) which is well comparable to the results obtained in the present study.

Influence of carbon and nitrogen was studied on the production of the pigment as well as the biomass. Additional maltose, as carbon source in the medium, enhanced the pigment production. Interference of carbon source with biosynthesis of many secondary metabolites may account for such enhancement (Demain 1986). The better pigmentation and growth have been observed in *Monascus* spp. when maltose is used along with other sugars (Omamor et al. 2008). Singgih et al. (2015) reported carotenoid production by *Neurospora intermedia N*-1 in presence of 2% w/v maltose as carbon source. Pradeep and Pradeep (2013) recorded maximum pigmentation by *Fusarium moniliforme* on adding glucose in the medium.

No additional nitrogen source in PD broth could enhance pigment production by GBPI_P155, although yeast extract resulted in enhancement in fungal biomass. C/N ratio plays a significant role in pigment production by filamentous fungi (Gmoser et al. 2017). In the present study, additional nitrogen source during optimisation might have resulted in disturbance of C/N ratio which decreased the final pigment production. Cho et al. (2002) and Gunasekaran and Poorniammal (2008) reported decrease in pigment production by *Paecilomyces sinclairii* and *Penicillium* sp., respectively, on increase in C/N ratio. Mineral salts, containing different cations, are also known to enhance pigment production (Premalatha et al. 2012; Pradeep and Pradeep 2013). In the present study, although potassium and magnesium salts facilitated pigment production, but not at par to the medium without such salts. These salts acted as inhibitors blocking release of the pigments by GBPI_P155.

FT-IR spectral pattern of extracted orange pigment in chloroform revealed possibility for the presence of carotenoid in the fungal extract. C=C functional group, determined through FT-IR, is a conjugated double bond which is basically present in carotenoid structure and acts as chromophore for light absorption, giving these compounds an attractive bright yellow to red colour (Gmoser et al. 2017). FT-IR spectra of other carotenoids have been reported in Lόránd et al. (2002) which showed similarity with the IR spectrum in the present study. The observation on a difference in fingerprint region of the IR spectrum indicated a possibility of different derivative of carotenoid. Following LC/MS of TLC-separated fractions, two derivatives namely tangeraxanthin and 4-ketonostoxanthin were identified. Tangeraxanthins are naturally occurring retro-apo carotenoids (Butnariu 2016). Another carotenoid (4-ketonostoxanthin 3′-sulphate), similar to 4-ketonostoxanthin, was also reported earlier by Yokoyama et al. (1996) in a marine bacterium. Compounds other than carotenoids such as anthracene and flavonoids were also found to be present in different fractions by the fungal isolate GBPI_P155. Mixture of carotenoids and other pigments produced by *Penicillium* sp., in the present study, might be related to their stress management process to cope up with UV radiation in higher altitudes. Moreover, these pigments may be harvested for potential uses after further screening for their toxic effects, if any. Mapari et al. (2010) have emphasised on potentially safe polyketide pigment produced by the filamentous fungus, suggesting fungal polyketide pigments, such as carotenoids, as natural food colourant.

GBPI_P155 produced mixture of carotenoids as well as some antibiotics (depicted by LC/MS data) which showed activity against bacteria and actinobacteria. In an earlier report by Patil et al. (2015), pigment produced by *P. purpurogenum* showed antimicrobial activity against *Pseudomonas aeruginosa* and *Staphylococcus aureus. Penicillium* sp. isolated by Saravanan and Radhakrishnan (2016) showed antimicrobial activity towards *B. subtilis, P. aeruginosa* and *E*. *coli* exhibiting diversified array of secondary metabolites possessing several important applications. Overall, the present study shows that the water insoluble orange pigment produced by the GBPI_P155 majorly contained derivatives of carotenoids along with the presence of anthracene and flavonoids and other compounds.

## 5. Conclusion

Pigments obtained from filamentous fungi are valuable bioactive compounds having great market demands for replacing synthetic pigments. Natural pigments from fungal sources have major advantage of their independent supply irrespective of any season. *Penicillium* sp. (GBPI_P155) appears to be a promising candidate for carotenoid production which might be due to the stress preventive mechanism possessed by the fungus to cope up with the extreme low-temperature environment prevailed under the mountain ecosystem of Himalaya. The antimicrobial property of pigments produced by GBPI_P155 provides an advantage of using them for several purposes such as dying, food colourant and for preventing any possible contamination.
