# Supplementary material for: Optimisation and characterisation of the orange pigment produced by a cold adapted strain of Penicillium sp. (GBPI_P155) isolated from mountain ecosystem
Source: Mycology. 2018 Jan 9;9(2):81–92. doi: 10.1080/21501203.2017.1423127 (PMC6059051; doi:10.1080/21501203.2017.1423127)
Supplement: Supplemetary_data.docx [file TMYC_A_1423127_SM9185.docx]

**Supplementary Data**

**Table S1.** Compounds of fraction A analysed by LC/MS

| **S.No.** | **Mass** | **Compound** | **Ion** | **Formula** |
| --- | --- | --- | --- | --- |
| 1 | 133.09 | No metabolite | - | - |
| 2 | 180.09 | N-Piperidin-4-ylurea--hydrogen chloride (1/1) | [M+H]^+^ | C_6_H_14_ClN_3_O |
| 3 | 274.30 | No metabolite |  |  |
| 4 | 365.18 | Ala Gln Phe | [M+H]^+^ | C_17_H_24_N_4_O_5_ |
| 5 | 453.39 | 2-Hydroxy-24-keto-octacosanolide | [M+H]^+^ | C_28_H_52_O_4_ |
| 6 | 457.20 | Flunisolide | [M+Na]^+^ | C_24_H_31_FO_6_ |
| 7 | 499.25 | **Sophoraisoflavanone C** | [M+Na]^+^ | C_30_H_36_O_5_ |
| 8 | 507.32 | **Tangeraxanthin** | [M+Na]^+^ | C_34_H_44_O_2_ |
| 9 | 601.54 | Oxalic acid--N''-tetradecylguanidine (1/2) | [M+H]^+^ | C_32_H_68_N_6_O_4_ |
| 10 | 679.58 | 1,1'-(4-methylbenzene-1,3-diyl)bis(3-hexadecylurea) | [M+Na]^+^ | C_41_H_76_N_4_O_2_ |

**Table S2.** Compounds of fraction B analysed through LC/MS

| **S.No.** | **Mass** | **Compound** | **Ion** | **Formula** |
| --- | --- | --- | --- | --- |
| 1 | 180.09 | N-Piperidin-4-ylurea--hydrogen chloride (1/1) | [M+H]^+^ | C_6_H_14_ClN_3_O |
| 2 | 235.15 | **2,3,9,10-tetramethyl-anthracene** | [M+H]^+^ | C_18_H_18_ |
| 3 | 261.13 | **4-methyl-3,4-dihydro-2H-benzo[a]anthracen-1-one** | [M+H]^+^ | C_19_H_16_O |
| 4 | 333.18 | Mestranol or Cannabinol | [M+Na]^+^ | C_21_H_26_O_2_ |
| 5 | 365.18 | Ala Gln Phe | [M+H]^+^ | C_17_H_24_N_4_O_5_ |
| 6 | 453.39 | 2-Hydroxy-24-keto-octacosanolide | [M+H]^+^ | C_28_H_52_O_4_ |
| 7 | 457.20 | Flunisolide | [M+Na]^+^ | C_24_H_31_FO_6_ |
| 8 | 507.32 | **Tangeraxanthin** | [M+Na]^+^ | C_34_H_44_O_2_ |
| 9 | 601.54 | Oxalic acid--N''-tetradecylguanidine (1/2) | [M+H]^+^ | C_32_H_68_N_6_O_4_ |
| 10 | 607.36 | Phe Gln Lys Lys Gly | [M+H]^+^ | C_28_H_46_N_8_O_7_ |
| 11 | 637.38 | 4-Ketonostoxanthin | [M+Na]^+^ | C_40_H_54_O_5_ |
| 12 | 872.64 | PS(20:0/22:2(13Z,16Z)) | [M+H]^+^ | C_48_H_90_NO_10_P |
| 13 | 960.71 | No metabolite | - | - |

**Table S3.** Compound of fraction C analysed by LC/MS

| **S.No.** | **Mass** | **Compound** | **Ion** | **Formula** |
| --- | --- | --- | --- | --- |
| 1 | 133.09 | No metabolite | - | - |
| 2 | 180.09 | N-Piperidin-4-ylurea--hydrogen chloride (1/1) | [M+H]^+^ | C_6_H_14_ClN_3_O |
| 3 | 274.30 | No metabolite | - | - |
| 4 | 301.17 | 4-[[1-(morpholin-4-ylmethyl)-4,5-dihydroimidazol-2-yl]sulfanylmethyl]morpholine | [M+H]^+^ | C_13_H_24_N_4_O_2_S |
| 5 | 365.18 | Ala Gln Phe | [M+H]^+^ | C_17_H_24_N_4_O_5_ |
| 6 | 453.39 | 2-Hydroxy-24-keto-octacosanolide | [M+H]^+^ | C_28_H_52_O_4_ |
| 7 | 457.20 | Flunisolide | [M+Na]^+^ | C_24_H_31_FO_6_ |
| 8 | 499.25 | **Sophoraisoflavanone C** | [M+Na]^+^ | C_30_H_36_O_5_ |
| 9 | 507.32 | **Tangeraxanthin** | [M+Na]^+^ | C_34_H_44_O_2_ |
| 10 | 601.54 | Oxalic acid--N''-tetradecylguanidine (1/2) | [M+H]^+^ | C_32_H_68_N_6_O_4_ |
| 11 | 872.64 | PS(20:0/22:2(13Z,16Z)) | [M+H]^+^ | C_48_H_90_NO_10_P |
| 12 | 960.70 | No metabolite | - | - |
